# Zilucoplan for Successful Early Weaning From Mechanical Ventilation and Avoiding Tracheostomy in an 85-Year-Old Woman Experiencing Myasthenic Crisis: A Case Report

**DOI:** 10.7759/cureus.80203

**Published:** 2025-03-07

**Authors:** Saki Ito, Takamichi Sugimoto, Hiroyuki Naito, Shiro Aoki, Masahiro Nakamori, Takafumi Miyachi, Yu Yamazaki, Hirofumi Maruyama

**Affiliations:** 1 Department of Clinical Neuroscience and Therapeutics, Hiroshima University, Hiroshima, JPN; 2 Department of Neurology, National Hospital Organization Yanai Medical Center, Yanai, JPN

**Keywords:** comorbidities, complications, myasthenic crisis, tracheostomy, weaning failure, zilucoplan

## Abstract

Myasthenic crisis is a life-threatening exacerbation of myasthenia gravis leading to respiratory failure, while zilucoplan is a C5 complement inhibitor that prevents complement-mediated destruction of the neuromuscular junction, thereby helping to restore muscle function and respiration. This case report describes an 85-year-old woman with late-onset myasthenia gravis who developed myasthenic crisis triggered by aspiration while eating. Initial treatment with Intravenous immunoglobulin and intravenous methylprednisolone showed limited efficacy, making weaning from mechanical ventilation challenging. Due to her advanced age, diabetes, and heart failure, plasmapheresis was considered high-risk. Zilucoplan, a C5 inhibitor administered subcutaneously, was introduced as an adjunctive therapy on Day 11, leading to rapid 50% hemolytic complement activity level reduction and significant muscle strength improvement. Extubation was achieved within four days, avoiding tracheostomy. Add-on therapy of zilucoplan provided a safe and accessible treatment option for myasthenic crisis. Previous reports have demonstrated the benefits of other C5 inhibitors and neonatal Fc receptor inhibitors in refractory myasthenic crisis. This case supports the early addition of zilucoplan with conventional rescue therapies for myasthenic crisis may have enabled earlier extubation and the avoidance of tracheostomy. Further studies are needed to determine the universal applicability of add-on therapies in myasthenic crisis, considering comorbidities and complications.

## Introduction

Intravenous immunoglobulin (IVIg), plasmapheresis, and intravenous methylprednisolone (IVMP) provide rapid relief of symptoms associated with acute exacerbation of myasthenia gravis (MG) [1]. Since researchers first reported the use of eculizumab in myasthenic crisis, characterized by respiratory insufficiency necessitating intubation and assisted ventilation in 2018, several reports have revealed the use of C5 inhibitors or Fc receptor inhibitors in combination therapy [2]. Zilucoplan, a peptide-based C5 inhibitor, binds to complement component C5, preventing its cleavage and subsequent formation of the membrane attack complex. It is administered via daily subcutaneous injections, making it an accessible option for adjunctive rescue therapy. Zilucoplan is a daily subcutaneous injection, whereas eculizumab and ravulizumab are administered as weekly or monthly intravenous infusions. Although add-on therapy with eculizumab and ravulizumab has been reported in cases of myasthenic crisis, there have been no previous reports of add-on therapy using zilucoplan. We report a case of myasthenic crisis in a patient with late-onset MG for whom the addition of zilucoplan to IVIg and IVMP allowed early weaning from mechanical ventilation, thereby avoiding the need for tracheostomy.

## Case presentation

A slender 83-year-old woman with type 1 diabetes who was positive for insulin antibodies, had diabetic neuropathy, chronic heart failure, paroxysmal tachycardia, atrial fibrillation, and hepatitis C, and had previously undergone gastrectomy for gastric cancer, and had a history of lumbar spinal canal stenosis, began experiencing bilateral lower extremity muscle weakness in May 2023, which persisted even after treatment for the spinal condition, leading her to use a cane. She began experiencing shortness of breath and developed fatigable ptosis of the right eye in August 2024. One month later, complete ptosis of the right eye was noted. She began choking during meals and developed diplopia. Her family physician tested her for anti-acetylcholine receptor (AChR) antibodies, for which she was positive (antibody titer: 210 nmol/L; reference value: ≤ 0.2 nmol/L), and she was referred to our department in October 2024 at age 85.

On examination, she exhibited complete ptosis of the right eye at rest. When her eyelid was passively lifted, she experienced diplopia immediately after gazing to the left. She also reported occasional dysphagia, had mild bilateral deltoid muscle weakness, and displayed gait instability. She was diagnosed with late-onset generalized MG. Tacrolimus (2 mg/day) was initiated on the same day. However, she developed a fever and became unresponsive shortly thereafter, leading to emergency transport to our hospital.

**Figure 1:**
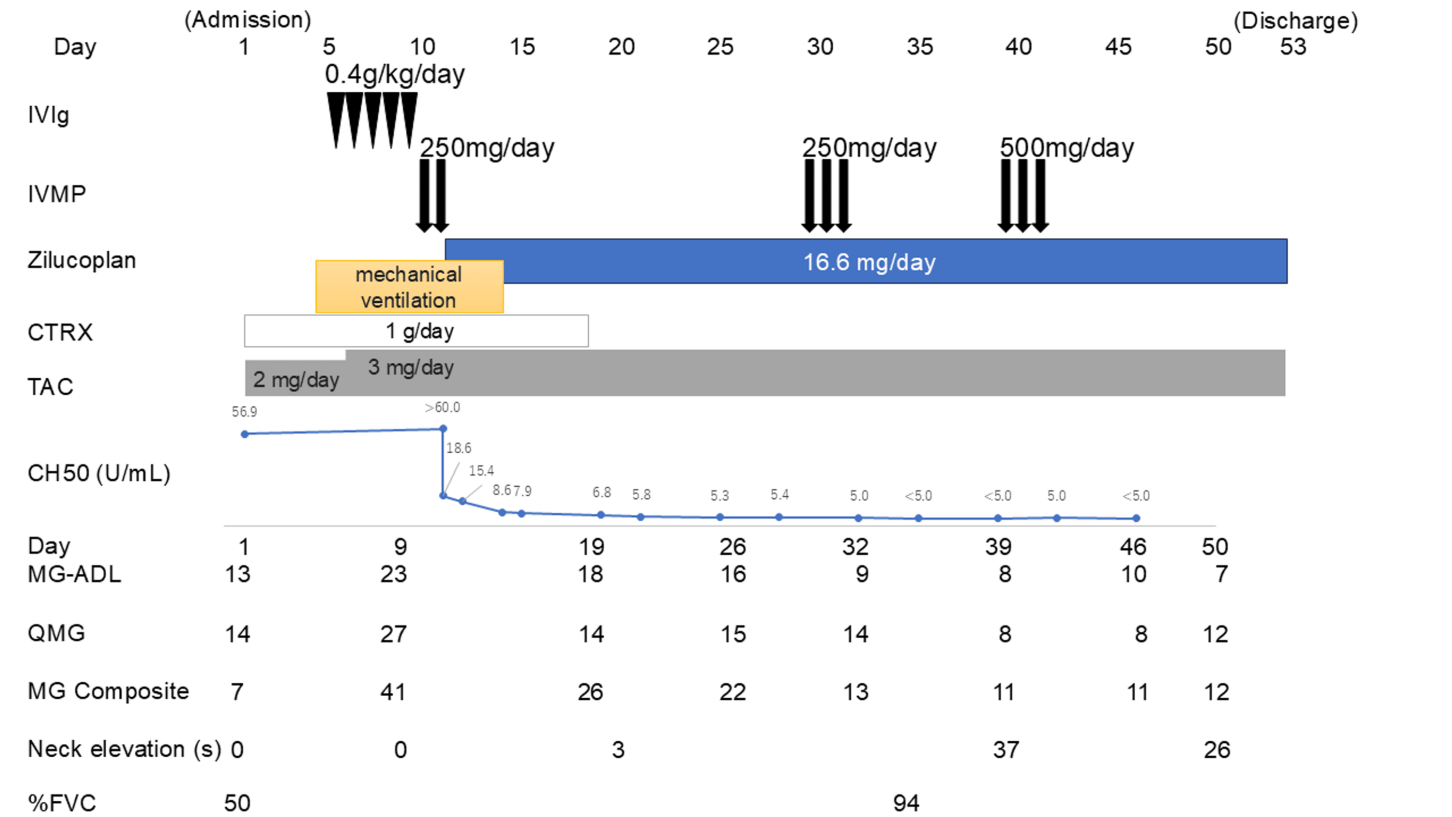
Clinical course of an 85-year-old female patient experiencing a myasthenic crisis who received zilucoplan as an add-on to conventional rescue therapy The clinical course from admission to discharge is illustrated, showing the progression from initial symptoms to respiratory deterioration, intubation, and treatment with IVIg, IVMP, tacrolimus, and zilucoplan. The introduction of zilucoplan led to a rapid decrease in CH50 levels and, together with other therapies, facilitated early extubation, contributing to the patient’s overall recovery and successful discharge. Based on the Myasthenia Gravis Composite Scale [3]. IVIg: intravenous immunoglobulin; IVMP: intravenous methylprednisolone; CTRX: ceftriaxone; TAC: tacrolimus; CH50: 50% hemolytic complement activity; MG-ADL: myasthenia gravis-activities of daily living scale; QMG: quantitative myasthenia gravis scale; MG composite: myasthenia gravis composite scale; %FVC: percent forced vital capacity

The clinical course from admission to discharge is summarized in Figure 1.

The patient presented with a body temperature of 39.0 degrees Celsius and a relatively low oxygenation level, and chest CT revealed infiltrative shadows in the left lung field, leading to a diagnosis of aspiration pneumonia. Ceftriaxone (1 g/day) was initiated. Pyridostigmine was not used in this case from admission to discharge, as it is not sufficiently effective in severe cases and carries a risk of promoting aspiration due to increased salivation. There were no findings suggestive of thymoma.

She communicated well at hospital admission. On Day 1, although she was unable to lift her head (the neck remained at 0 degrees) while positioned supine, she was able to maintain her upper limbs in the elevated position for 240 seconds while seated. She was able to consume soft meals. The morning scores for the Quantitative Myasthenia Gravis (QMG) scale [4] and Myasthenia Gravis Activities of Daily Living (MG-ADL) scale [5] were 14 and 13 points, respectively. Laboratory tests revealed a glycated hemoglobin (HbA1c) of 7.6% and an NT-proBNP of 4602 pg/mL, with no indications of renal or hepatic dysfunction. She was observed for the next three days, and on Day 4, her food intake decreased while aspiration of soft foods became more frequent, leading to a diagnosis of Myasthenia Gravis Foundation of America classification IVb [4] after dinner. The Manual Muscle Testing (MMT) score was four for the bilateral deltoids and five for the iliopsoas muscles, with mild gait instability.

**Figure 2:**
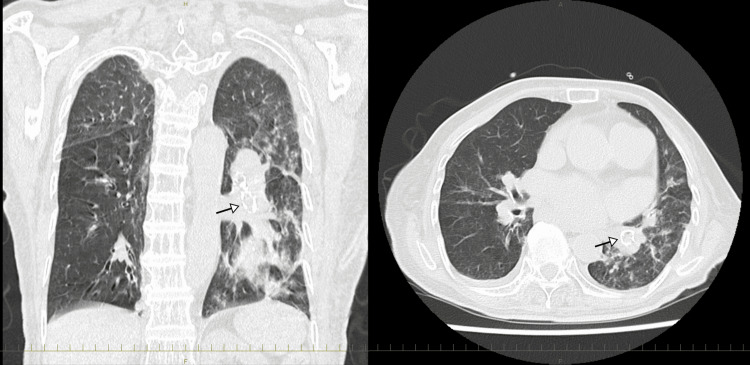
The CT image showing sputum retention in the left lower lobar bronchus Aspiration-related hypoxemia and choking led to sputum retention (arrow), which triggered the need for endotracheal intubation and mechanical ventilation.

On the night of Day 4, her oxygen saturation (SpO_2_) decreased before the insertion of a nasogastric tube, which was attributed to poor oxygenation due to aspiration while eating, leading to choking. A CT scan confirmed sputum retention in the left lower lobar bronchus, as shown in Figure 2.

As a result, she underwent endotracheal intubation and was started on mechanical ventilation. Intravenous immunoglobulin (0.4 g/kg/day) was started on Day 5 and administered for five days thereafter. Considering the potential use of a C5 inhibitor in the near future, a meningococcal (Groups A, C, Y, W) conjugate vaccine was administered on Day 5. Tacrolimus was increased to 3 mg/day on Day 6. On Days 10 and 11, IVMP (250 mg) was administered, but respiratory function did not improve, so weaning was not possible. We chose this dosage to avoid strong initial exacerbation, delayed recovery, and increased tracheostomy risk. Additionally, even with this dosage, it was expected to sufficiently improve muscle strength.

On Day 11, zilucoplan (16.6 mg/day, administered subcutaneously) was started. Her 50% hemolytic complement activity (CH50) levels rapidly decreased from >60 U/mL to 18.6 U/mL on the same day. On Day 13, the patient was able to stand with support and move her upper limbs easily; thus, weaning was considered possible. On Day 14, spontaneous breathing led to satisfactory oxygenation, so extubation was performed. Ceftriaxone 1 g/day was given until Day 18, for two weeks from the initiation of the meningococcal vaccine, to prevent meningococcal infection. On Day 34, we confirmed that the percent forced vital capacity had improved to 94%, compared to 50% at admission. On Day 35, 25 days after the start of zilucoplan, her CH50 levels had dropped below the detection limit. Two additional cycles of IVMP (250 mg/day for three days and 500 mg/day for three days) were administered. As shown in Figure 1, swallowing function and the ability to maintain neck elevation gradually improved, along with overall muscle strength with the IVMP and zilucoplan treatment.

On Day 53, the patient had no issues swallowing, was able to walk short distances without assistance, and was discharged home. At discharge, her QMG score was 12, and her MG-ADL score was seven.

## Discussion

In this case, myasthenic crisis was triggered by aspiration, and the patient showed poor short-term responsiveness to IVIg and IVMP. However, the addition of zilucoplan enabled early extubation. Shortly after the administration of IVIg and IVMP, there was little improvement in chest wall movement, making weaning difficult. Following the introduction of zilucoplan, chest wall movement improved, and extubation was successful within four days from the use of zilucoplan, avoiding the need for tracheostomy.

Weaning failure is characterized by a patient’s inability to maintain spontaneous breathing while ventilatory support is gradually decreased. According to a report by Neumann et al. [6], weaning failure was observed in 64% of patients with myasthenia gravis. Risk factors include advanced age; the presence of comorbidities such as hypertension, diabetes, and cardiac disease; and complications such as atelectasis, pneumonia, cardiopulmonary resuscitation, and sepsis, in addition to the choice of treatment modality. Plasmapheresis has been reported to be associated with a lower risk of weaning failure compared to IVIg.

Our patient had multiple risk factors for weaning failure, including advanced age, type 1 diabetes, chronic heart failure, and aspiration pneumonia. Treatment with IVIg and IVMP was not immediately effective, suggesting that extubation would require additional time. Consequently, the risk of weaning failure and the need for tracheostomy were significant. In our case, therapeutic plasmapheresis posed a potential risk of hemodynamic instability due to the patient’s advanced age and chronic heart failure. Therefore, zilucoplan, which is administered subcutaneously and associated with minimal cardiac burden, was chosen as an adjunct treatment. Additionally, zilucoplan is a daily injectable preparation, eliminating the need for supplemental therapy often required with eculizumab or ravulizumab in conjunction with therapeutic plasmapheresis or IVIg. This flexibility made zilucoplan an accessible option for treatment in this case.

Since 2018, there have been reports of cases in which the addition of eculizumab or efgartigimod as rescue therapy for myasthenic crisis requiring mechanical ventilation resulted in symptom improvement. More recently, a case of refractory myasthenic crisis treated with ravulizumab has been reported (Table 1) [2, 7-12].

**Table 1:** Cases of myasthenic crisis in which a C5 inhibitor or FcRn inhibitor was added as an add-on to conventional rescue therapies FcRn: neonatal Fc receptor; MG: myasthenia gravis; AChR: acetylcholine receptor; EO: early-onset; LO: late-onset; TA: thymoma-associated; IVIg: intravenous immunoglobulin; IVMP: intravenous methylprednisolone

Author	Age at myasthenic crisis/sex/MG-subtype (antibody)	Type of rescue therapies before the add-on therapy	Reason for add-on therapy	Add-on drug /Start date of add-on therapy	Days from intubation to extubation /Days from initiation of add-on therapy to extubation	Comorbidities or complications
Yeo [2]	79/F/Seronegative	Plasmapheresis/IVIg	Previous good responses to eculizumab and rituximab for atypical hemolytic uremic syndrome, and low CD20 expression	Eculizumab /day 25 after intubation	Within 6 weeks/Approximately within 3 weeks	Atypical hemolytic uremic syndrome, bronchiectasis
Usman, case 1 [7]	24/F/EO (AChR)	Plasmapheresis/IVIg/Rituximab	Refractory to the rescue therapy	Eculizumab/week 11 after intubation	Approximately 12 weeks/Within 1 week	Intestinal perforation
Usman, case 2 [7]	77/M/LO (AChR)	Plasmapheresis/IVIg	Refractory to the rescue therapy	Eculizumab/Day 22 after intubation	23 days/2 days	None
Usman, case 3 [7]	56/M/LO (AChR)	Plasmapheresis/IVIg	Refractory to the rescue therapy	Eculizumab /Week 4 after admission	Not mentioned details /Not mentioned details (remained on intermittent noninvasive ventilation through tracheostomy)	Intestinal perforation, aspiration pneumonia, adrenal insufficiency
Vinciguerra [8]	74/M/LO (AChR)	IVIg	No immediate response to the rescue therapy, systemic conditions such as pneumonia and sepsis	Eculizumab/1 month after intubation	No details mentioned but approximately within 35 days/Within 5 days	Type 2 diabetes mellitus, pneumonia, sepsis
Watanabe [9]	54/F/TA (AChR)	Plasmapheresis/IVIg	Worsening despite the rescue therapy	Efgartigimod/Soon after intubation	56 days/A period almost equal to the duration of intubation	Lumbar disc herniation
Omar [10]	57/M/LO (AChR)	Plasmapheresis	Worsening despite the rescue therapy	Efgartigimod/Not mentioned	Not mentioned/Not mentioned	A mechanical heart valve, depression, anxiety disorder, chronic somatoform pain disorder, arterial hypertension, and diabetes mellitus with polyneuropathy
Ohara [11]	70/F/LO (AChR)	None	Shortage of IVIg supply, difficulty in creating blood access	Efgartigimod/Day 1 after intubation	15 days/15 days	None
Uchi [12]	71/F/LO (AChR)	IVIg/Efgartigimod/IVMP	Refractory to the available therapies	Ravulizumab /Day 20 after the intubation	40 days /21 days	Type 1 diabetes mellitus
This case	85/F/LO (AChR)	IVIg/IVMP	No immediate response to the rescue therapy, a potential risk of hemodynamic instability due to the patient’s advanced age and chronic heart failure, no need for supplemental therapy for other C5 inhibitors or IVIg	Zilucoplan/Day 8 after the intubation	11 days/4 days	Type 1 diabetes mellitus with polyneuropathy, chronic heart failure, paroxysmal tachycardia, atrial fibrillation, underwent gastrectomy for gastric cancer, hepatitis C, aspiration pneumonia

The cases reported involved patients ranging in age from 24 to 85 years and were predominantly cases of thymoma-associated MG or late-onset MG. In many cases shown in Table 1, C5 inhibitors or neonatal Fc receptor (FcRn) inhibitors were used as add-on therapy due to worsening or a refractory or partial immediate response to conventional rescue therapy. Ohara et al. described the early introduction of efgartigimod as a rescue therapy, which was administered concurrently with IVMP [11]. Similarly, in this case, zilucoplan was administered relatively early. In particular, patients for whom C5 inhibitors were administered as add-on therapy were often weaned from mechanical ventilation early after its initiation. Early use of add-on therapies for C5 inhibitors should be explored in future studies. Indeed, whether add-on therapy is universally applicable to all cases of myasthenic crisis, regardless of the presence or absence of comorbidities or complications, remains unclear and requires further investigation.

On the day zilucoplan was initiated, the CH50 level decreased from >60 U/mL to 18.6 U/mL. In the report by Uchi et al., which used ravulizumab for myasthenic crisis, the patient's serum hemolytic complement activity (CH50) levels were between 17.8 and 20.0 U/L after two cycles of ravulizumab treatment [12]. The degree and course of CH50 reduction following eculizumab or ravulizumab administration may vary among patients. Although the CH50 level did not fall below the lower limit of normal in our case, the patient exhibited significant improvement in muscle strength shortly after its administration, suggesting that achieving an undetectable CH50 level may not be an absolute requirement for therapeutic efficacy. As this issue has not been sufficiently investigated, further studies are needed to clarify the relationship between CH50 levels and clinical improvement.

In this case, IVIg and IVMP alone did not lead to rapid symptom improvement, but the addition of zilucoplan facilitated early extubation, likely through enhancing muscle strength through complement inhibition. However, whether this improvement was due to zilucoplan alone or a synergistic effect with IVIg and IVMP remains unclear. Further research, including studies on add-on therapy and monotherapy with zilucoplan, is needed to determine its role in the management of myasthenic crisis.

## Conclusions

We noted that the early addition of zilucoplan with conventional rescue therapies for myasthenic crisis may have enabled earlier extubation and the avoidance of tracheostomy. This case suggests that a C5 inhibitor could be a valuable therapeutic option, even in older patients with multiple comorbidities who face significant challenges in recovery. Further studies, such as the accumulation of case reports and clinical trials, are needed to determine the universal applicability of add-on therapies in myasthenic crisis, considering comorbidities and complications.
